# *catena*-Poly[[[tetra­aqua­praseo­dym­ium(III)]-di-μ-nicotinato-κ^2^ *O*:*N*;κ^2^ *O*:*N*-disilver(I)-di-μ-nicotinato-κ^2^ *N*:*O*;κ^2^ *N*:*O*] perchlorate monohydrate]

**DOI:** 10.1107/S1600536809001718

**Published:** 2009-01-17

**Authors:** Biao Guan, Chao-Hua Zhang, Wen-Dong Song

**Affiliations:** a Laboratory and Facility Management Division, Guangdong Ocean University, Zhanjiang 524088, People’s Republic of China; b School of Food Science and Technology, Guangdong Ocean University, Zhanjiang 524088, People’s Republic of China; c College of Science, Guangdong Ocean University, Zhanjiang 524088, People’s Republic of China

## Abstract

In the title compound, {[Ag_2_Pr(C_6_H_4_NO_2_)_4_(H_2_O)_4_]ClO_4_·H_2_O}_*n*_, the Pr^III^ atom, lying on a twofold rotation axis, has a distorted square-anti­prismatic coordination geometry, defined by four O atoms from four nicotinate (nic) ligands and four water mol­ecules. The Ag^I^ atom is coordinated in an almost linear fashion by two pyridyl N atoms from two nicotinate ligands. The linear coordination is augmented by weak inter­actions with three O atoms from one perchlorate anion, one uncoordinated water mol­ecule and one carboxyl­ate group. Two Pr atoms link two {Ag(nic)_2_}^+^ units into a ring, which is further extended into an infinite zigzag chain by sharing the Pr atoms. These chains are further connected into a three-dimensional network *via* weak Ag⋯O inter­actions, O—H⋯O hydrogen bonds, Ag⋯Ag inter­actions [3.357 (2) Å] and π–π inter­actions between the pyridyl rings [centroid–centroid distance = 3.685 (4) Å].

## Related literature

For general background, see: Cheng *et al.* (2007*a* ▶,*b* ▶); Luo *et al.* (2006 ▶, 2007 ▶).
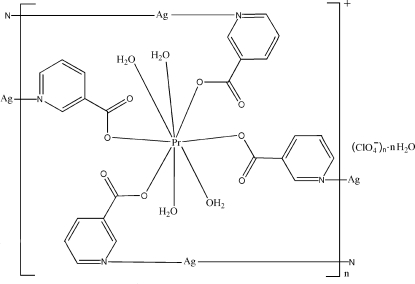


## Experimental

### 

#### Crystal data

[Ag_2_Pr(C_6_H_4_NO_2_)_4_(H_2_O)_4_]ClO_4_·H_2_O
*M* *_r_* = 1034.59
Orthorhombic, 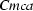

*a* = 35.396 (3) Å
*b* = 12.3733 (10) Å
*c* = 15.2324 (13) Å
*V* = 6671.2 (10) Å^3^
*Z* = 8
Mo *K*α radiation
μ = 2.76 mm^−1^
*T* = 273 (2) K
0.30 × 0.25 × 0.22 mm

#### Data collection

Bruker APEXII CCD diffractometer
Absorption correction: multi-scan (*SADABS*; Sheldrick, 1996 ▶) *T*_min_ = 0.453, *T*_max_ = 0.552
16336 measured reflections
3065 independent reflections
2478 reflections with *I* > 2σ(*I*)
*R*_int_ = 0.049

#### Refinement

*R*[*F* ^2^ > 2σ(*F* ^2^)] = 0.036
*wR*(*F* ^2^) = 0.097
*S* = 1.06
3065 reflections
227 parameters
27 restraints
H-atom parameters constrained
Δρ_max_ = 1.56 e Å^−3^
Δρ_min_ = −0.87 e Å^−3^

### 

Data collection: *APEX2* (Bruker, 2007 ▶); cell refinement: *SAINT* (Bruker, 2007 ▶); data reduction: *SAINT*; program(s) used to solve structure: *SHELXS97* (Sheldrick, 2008 ▶); program(s) used to refine structure: *SHELXL97* (Sheldrick, 2008 ▶); molecular graphics: *SHELXTL* (Sheldrick, 2008 ▶); software used to prepare material for publication: *SHELXTL*.

## Supplementary Material

Crystal structure: contains datablocks I, global. DOI: 10.1107/S1600536809001718/hy2177sup1.cif

Structure factors: contains datablocks I. DOI: 10.1107/S1600536809001718/hy2177Isup2.hkl

Additional supplementary materials:  crystallographic information; 3D view; checkCIF report

## Figures and Tables

**Table 1:** Selected bond lengths (Å)

Pr1—O3^i^	2.390 (14)
Pr1—O1*W*	2.477 (14)
Pr1—O2*W*	2.495 (14)
Pr1—O1	2.504 (13)
Ag1—N2	2.165 (18)
Ag1—N1	2.175 (18)
Ag1—O4^ii^	2.777 (16)
Ag1—O5	2.81 (3)
Ag1—O3*W*	2.90 (2)

Symmetry codes: (i) 
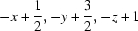
; (ii) 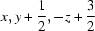.

**Table 2:** Hydrogen-bond geometry (Å, °)

*D*—H⋯*A*	*D*—H	H⋯*A*	*D*⋯*A*	*D*—H⋯*A*
O1*W*—H1*W*⋯O2^iii^	0.88	1.79	2.67 (2)	179
O1*W*—H2*W*⋯O4^iv^	0.97	1.68	2.63 (2)	163
O2*W*—H3*W*⋯O2^v^	1.00	1.77	2.77 (2)	176
O2*W*—H4*W*⋯O2^vi^	0.83	1.95	2.76 (2)	162
O3*W*—H5*W*⋯O1*W*^vii^	0.82	2.15	2.91 (2)	157

Symmetry codes: (iii) 
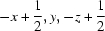
; (iv) 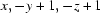; (v) 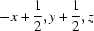; (vi) 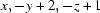; (vii) 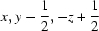.

**Fig. 1.**
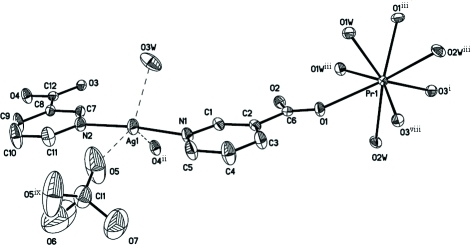
The asymmetric unit of the title compound, extended to show the Pr and Ag coordination environments. Displacement ellipsoids are drawn at the 50% probability level. [Symmetry codes: (i) 1/2 - x, 3/2 - y, 1 - z; (ii) x, 1/2 + y, 3/2 - z; (iii) 1/2 - x, y, 1/2 - z; (viii) x, 3/2 - y, -1/2 + z; (ix) -x, y, z.]

**Fig. 2.**
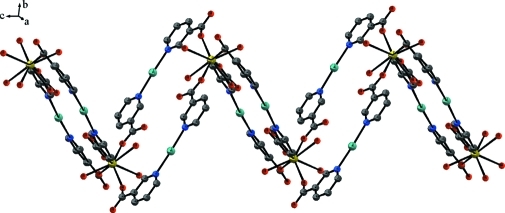
View of the zigzag chain in the title compound.

## supplementary crystallographic information

### Comment

Nicotinic acid is a multifunctional bridging ligand possessing of O and N donors, which can thus be utilized to construct lanthanide–transition heterometallic complexes, *via* carboxylate O atoms binding to lanthanides and N atoms binding to transition metal ions such as Ag^I^ or Cu^I^ (Cheng *et al.*, 2007*a*,b; Luo *et al.*, 2006, 2007). On the basis of above considerations, we chose nicotinic acid, Pr^III^ and Ag^I^ metal ions as building blocks. A new one-dimensional 4 d–4f coordination polymer was obtained from the hydrothermal treatment of Pr_6_O_11_, AgNO_3_, perchloric acid and nicotinic acid in water.

In the title compound (Fig. 1), the Pr^III^ atom, lying on a twofold rotation axis, has a distorted square-antiprismatic coordination geometry, defined by four O atoms from four nicotinate (nic) ligands and four water molecules. The perchlorate anion lies on a mirror plane and the uncoordinated water molecule lies on a twofold rotation axis. The Ag^I^ atom is coordinated in an almost linear fashion by two pyridyl N atoms from two nic ligands. The linear coordination are augmented by weak Ag···O interactions with one O atom from the ClO_4_^-^ anion, one O atom from the uncoordinated water molecule and one carboxylate O atom from the nic ligand (Table 1). The Ag atom also exhibits an argentophilic interaction, with an Ag···Ag distance of 3.357 (1) Å. The pyridyl rings of the nic ligands coordinating to the Ag atom are almost coplanar and have a dihedral angle of 1.74 (2)°. Two Pr atoms link two Ag(nic)_2_^+^ units into a ring, which are further extended into an infinite zigzag chain by sharing the common Pr atoms (Fig. 2). These chains are further connected into a three-dimensional network *via* the weak Ag···O interactions, O—H···O hydrogen bonds (Table 2), weak Ag···Ag interactions and π–π interactions occurring between the pyridyl rings of neighboring nic ligands [centroid–centroid distance = 3.685 (4) Å].

### Experimental

A mixture of Pr_6_O_11_ (0.170 g, 0.5 mmol), AgNO_3_ (0.169 g, 1 mmol), nicotinic acid (0.123 g, 1 mmol), HClO_4_ (0.12 ml) and H_2_O (10 ml) was placed in a 23 ml Teflon-lined reactor, which was heated to 433 K for 3 d and then cooled to room temperature at a rate of 10 K h^-1^. The pale-purple plate crystals obtained were washed with water and dried in air (yield 46% based on Pr).

### Refinement

H atoms on C atoms were positioned geometrically and treated as riding on the parent C atoms, with C—H = 0.93 Å and *U*_iso_(H) = 1.2*U*_eq_(C). H atoms of water molecules were located in difference Fourier maps and fixed in the refinements, with *U*_iso_(H) = 1.5*U*_eq_(O). The highest residual electron density was found 1.09 Å from atom Pr1 and the deepest hole 0.76 Å from atom Cl1.

### Crystal data

**Table:**

[Ag_2_Pr(C_6_H_4_NO_2_)_4_(H_2_O)_4_]ClO_4_·H_2_O	*F*(000) = 4032
*M**_r_* = 1034.59	*D*_x_ = 2.060 Mg m^−^^3^
Orthorhombic, *C**m**c**a*	Mo *K*α radiation, λ = 0.71073 Å
Hall symbol: -C 2bc 2	Cell parameters from 3121 reflections
*a* = 35.396 (3) Å	θ = 1.4–28°
*b* = 12.3733 (10) Å	µ = 2.76 mm^−^^1^
*c* = 15.2324 (13) Å	*T* = 273 K
*V* = 6671.2 (10) Å^3^	Plate, pale purple
*Z* = 8	0.30 × 0.25 × 0.22 mm

### Data collection

**Table:**

Bruker APEXII CCD diffractometer	3065 independent reflections
Radiation source: fine-focus sealed tube	2478 reflections with *I* > 2σ(*I*)
graphite	*R*_int_ = 0.049
φ and ω scans	θ_max_ = 25.2°, θ_min_ = 2.2°
Absorption correction: multi-scan (*SADABS*; Sheldrick, 1996)	*h* = −28→42
*T*_min_ = 0.453, *T*_max_ = 0.552	*k* = −13→14
16336 measured reflections	*l* = −18→18

### Refinement

**Table:**

Refinement on *F*^2^	Primary atom site location: structure-invariant direct methods
Least-squares matrix: full	Secondary atom site location: difference Fourier map
*R*[*F*^2^ > 2σ(*F*^2^)] = 0.036	Hydrogen site location: inferred from neighbouring sites
*wR*(*F*^2^) = 0.097	H-atom parameters constrained
*S* = 1.06	*w* = 1/[σ^2^(*F*_o_^2^) + (0.0479*P*)^2^ + 31.5675*P*] where *P* = (*F*_o_^2^ + 2*F*_c_^2^)/3
3065 reflections	(Δ/σ)_max_ < 0.001
227 parameters	Δρ_max_ = 1.56 e Å^−^^3^
27 restraints	Δρ_min_ = −0.86 e Å^−^^3^

### Fractional atomic coordinates and isotropic or equivalent isotropic displacement parameters (Å^2^)

**Table:**

	*x*	*y*	*z*	*U*_iso_*/*U*_eq_	
Pr1	0.2500	1.08315 (12)	0.2500	0.0216 (5)	
Ag1	0.11088 (6)	0.60988 (15)	0.56463 (12)	0.0439 (6)	
C2	0.1570 (6)	0.8876 (17)	0.4208 (13)	0.031 (5)	
C6	0.1963 (6)	0.9230 (16)	0.3979 (13)	0.027 (4)	
C3	0.1260 (7)	0.943 (2)	0.3901 (17)	0.048 (6)	
H3	0.1292	1.0038	0.3555	0.058*	
C1	0.1508 (6)	0.7976 (18)	0.4725 (13)	0.033 (5)	
H1	0.1716	0.7607	0.4945	0.040*	
C4	0.0904 (8)	0.908 (2)	0.411 (2)	0.067 (9)	
H4	0.0692	0.9439	0.3899	0.081*	
C5	0.0867 (7)	0.816 (2)	0.4631 (18)	0.054 (7)	
H5	0.0625	0.7927	0.4782	0.065*	
N1	0.1163 (5)	0.7608 (15)	0.4925 (12)	0.040 (5)	
O1	0.2002 (4)	0.9976 (12)	0.3439 (9)	0.035 (3)	
O2	0.2235 (4)	0.8725 (12)	0.4344 (9)	0.033 (3)	
N2	0.1025 (5)	0.4681 (15)	0.6456 (12)	0.037 (4)	
C7	0.1323 (6)	0.4144 (16)	0.6770 (14)	0.032 (5)	
H7	0.1563	0.4385	0.6610	0.038*	
C11	0.0685 (8)	0.434 (2)	0.669 (2)	0.057 (8)	
H11	0.0474	0.4718	0.6489	0.069*	
Cl1	0.0000	0.6849 (10)	0.5898 (10)	0.080 (4)	
O7	0.0000	0.803 (3)	0.588 (4)	0.150 (19)	
O6	0.0000	0.647 (5)	0.685 (4)	0.25 (4)	
C8	0.1300 (6)	0.3255 (16)	0.7316 (13)	0.030 (5)	
C9	0.0945 (7)	0.292 (2)	0.7558 (19)	0.057 (8)	
H9	0.0914	0.2344	0.7941	0.069*	
C10	0.0631 (8)	0.346 (3)	0.723 (3)	0.082 (12)	
H10	0.0388	0.3232	0.7362	0.098*	
C12	0.1652 (6)	0.2676 (16)	0.7619 (13)	0.028 (4)	
O3	0.1962 (4)	0.3019 (12)	0.7341 (9)	0.032 (3)	
O4	0.1611 (4)	0.1878 (13)	0.8104 (11)	0.044 (4)	
O1W	0.2180 (4)	0.9435 (12)	0.1603 (9)	0.035 (4)	
H1W	0.2373	0.9195	0.1295	0.053*	
H2W	0.1996	0.8918	0.1807	0.053*	
O2W	0.2515 (5)	1.1653 (13)	0.3996 (10)	0.046 (4)	
H3W	0.2615	1.2394	0.4110	0.069*	
H4W	0.2454	1.1409	0.4486	0.069*	
O3W	0.1776 (8)	0.5000	0.5000	0.080 (10)	
H5W	0.1920	0.5010	0.4583	0.120*	
O5	0.0324 (9)	0.643 (3)	0.565 (3)	0.19 (2)	

### Atomic displacement parameters (Å^2^)

**Table:**

	*U*^11^	*U*^22^	*U*^33^	*U*^12^	*U*^13^	*U*^23^
Pr1	0.0227 (9)	0.0223 (8)	0.0199 (8)	0.000	0.0012 (6)	0.000
Ag1	0.0482 (12)	0.0376 (11)	0.0458 (11)	−0.0044 (9)	0.0028 (8)	0.0151 (8)
C2	0.035 (12)	0.033 (11)	0.024 (10)	0.001 (10)	0.006 (9)	0.001 (9)
C6	0.032 (12)	0.025 (10)	0.023 (10)	0.000 (9)	0.005 (9)	−0.004 (8)
C3	0.038 (14)	0.051 (15)	0.054 (15)	0.000 (12)	0.009 (12)	0.023 (12)
C1	0.034 (12)	0.038 (12)	0.028 (10)	−0.003 (10)	0.003 (9)	0.004 (10)
C4	0.035 (15)	0.07 (2)	0.09 (2)	0.003 (14)	0.004 (15)	0.043 (17)
C5	0.034 (14)	0.060 (17)	0.069 (18)	−0.005 (13)	0.012 (12)	0.023 (14)
N1	0.039 (11)	0.040 (11)	0.040 (10)	−0.002 (9)	0.006 (8)	0.014 (9)
O1	0.034 (8)	0.032 (8)	0.037 (8)	0.000 (7)	0.005 (6)	0.013 (7)
O2	0.029 (8)	0.038 (8)	0.031 (8)	0.000 (7)	0.000 (6)	0.005 (7)
N2	0.034 (11)	0.033 (10)	0.045 (11)	0.000 (8)	0.002 (8)	0.010 (8)
C7	0.030 (12)	0.028 (11)	0.037 (12)	−0.006 (9)	0.001 (9)	0.001 (9)
C11	0.037 (15)	0.054 (17)	0.08 (2)	0.006 (12)	0.003 (13)	0.029 (15)
Cl1	0.040 (6)	0.070 (8)	0.130 (11)	0.000	0.000	0.009 (7)
O7	0.17 (5)	0.09 (3)	0.19 (5)	0.000	0.000	0.04 (3)
O6	0.45 (13)	0.14 (5)	0.15 (6)	0.000	0.000	0.02 (4)
C8	0.030 (12)	0.024 (11)	0.036 (12)	−0.002 (9)	0.004 (9)	0.001 (9)
C9	0.034 (14)	0.054 (16)	0.08 (2)	−0.002 (12)	0.005 (13)	0.042 (15)
C10	0.029 (15)	0.08 (2)	0.13 (3)	0.000 (15)	0.004 (17)	0.06 (2)
C12	0.026 (11)	0.028 (11)	0.031 (11)	0.000 (9)	0.003 (8)	−0.005 (9)
O3	0.027 (8)	0.031 (8)	0.037 (8)	−0.005 (6)	0.002 (6)	−0.005 (6)
O4	0.031 (9)	0.044 (10)	0.056 (10)	0.005 (7)	0.007 (7)	0.023 (8)
O1W	0.029 (8)	0.035 (8)	0.041 (9)	−0.006 (6)	0.006 (7)	−0.009 (7)
O2W	0.071 (12)	0.044 (10)	0.024 (8)	−0.021 (9)	0.013 (8)	−0.010 (7)
O3W	0.047 (16)	0.15 (3)	0.040 (14)	0.000	0.000	−0.002 (17)
O5	0.07 (2)	0.14 (3)	0.35 (6)	0.04 (2)	0.07 (3)	0.12 (3)

### Geometric parameters (Å, °)

**Table:**

Pr1—O3^i^	2.390 (14)	N2—C11	1.32 (3)
Pr1—O1W	2.477 (14)	N2—C7	1.34 (3)
Pr1—O2W	2.495 (14)	C7—C8	1.38 (3)
Pr1—O1	2.504 (13)	C7—H7	0.9300
Ag1—N2	2.165 (18)	C11—C10	1.37 (4)
Ag1—N1	2.175 (18)	C11—H11	0.9300
Ag1—O4^ii^	2.777 (16)	Cl1—O5^iv^	1.31 (3)
Ag1—O5	2.81 (3)	Cl1—O5	1.31 (3)
Ag1—O3W	2.90 (2)	Cl1—O7	1.46 (4)
Ag1—Ag1^iii^	3.357 (2)	Cl1—O6	1.52 (5)
C2—C3	1.37 (3)	C8—C9	1.37 (3)
C2—C1	1.38 (3)	C8—C12	1.51 (3)
C2—C6	1.50 (3)	C9—C10	1.39 (4)
C6—O1	1.24 (2)	C9—H9	0.9300
C6—O2	1.27 (3)	C10—H10	0.9300
C3—C4	1.37 (4)	C12—O4	1.24 (2)
C3—H3	0.9300	C12—O3	1.25 (2)
C1—N1	1.34 (3)	O1W—H1W	0.88
C1—H1	0.9300	O1W—H2W	0.97
C4—C5	1.39 (4)	O2W—H3W	1.00
C4—H4	0.9300	O2W—H4W	0.83
C5—N1	1.33 (3)	O3W—H5W	0.82
C5—H5	0.9300		
			
O3^i^—Pr1—O3^v^	106.9 (7)	C3—C4—H4	120.8
O3^i^—Pr1—O1W	146.7 (5)	C5—C4—H4	120.8
O3^v^—Pr1—O1W	89.7 (5)	N1—C5—C4	123 (2)
O3^i^—Pr1—O1W^vi^	89.7 (5)	N1—C5—H5	118.7
O3^v^—Pr1—O1W^vi^	146.7 (5)	C4—C5—H5	118.7
O1W—Pr1—O1W^vi^	91.5 (7)	C5—N1—C1	118 (2)
O3^i^—Pr1—O2W	69.4 (5)	C5—N1—Ag1	122.9 (16)
O3^v^—Pr1—O2W	82.3 (5)	C1—N1—Ag1	119.1 (15)
O1W—Pr1—O2W	142.9 (5)	C6—O1—Pr1	140.4 (13)
O1W^vi^—Pr1—O2W	76.7 (5)	C11—N2—C7	118 (2)
O3^i^—Pr1—O2W^vi^	82.3 (5)	C11—N2—Ag1	122.3 (16)
O3^v^—Pr1—O2W^vi^	69.4 (5)	C7—N2—Ag1	119.9 (14)
O1W—Pr1—O2W^vi^	76.7 (5)	N2—C7—C8	124 (2)
O1W^vi^—Pr1—O2W^vi^	142.9 (5)	N2—C7—H7	117.8
O2W—Pr1—O2W^vi^	131.9 (7)	C8—C7—H7	117.8
O3^i^—Pr1—O1	139.0 (5)	N2—C11—C10	123 (2)
O3^v^—Pr1—O1	75.5 (5)	N2—C11—H11	118.7
O1W—Pr1—O1	72.5 (5)	C10—C11—H11	118.7
O1W^vi^—Pr1—O1	73.2 (5)	O5^iv^—Cl1—O5	122 (4)
O2W—Pr1—O1	70.5 (5)	O5^iv^—Cl1—O7	113.2 (18)
O2W^vi^—Pr1—O1	132.8 (5)	O5—Cl1—O7	113.2 (18)
O3^i^—Pr1—O1^vi^	75.5 (5)	O5^iv^—Cl1—O6	98 (2)
O3^v^—Pr1—O1^vi^	139.0 (5)	O5—Cl1—O6	98 (2)
O1W—Pr1—O1^vi^	73.2 (5)	O7—Cl1—O6	109 (3)
O1W^vi^—Pr1—O1^vi^	72.5 (5)	C9—C8—C7	117 (2)
O2W—Pr1—O1^vi^	132.8 (5)	C9—C8—C12	122.1 (19)
O2W^vi^—Pr1—O1^vi^	70.5 (5)	C7—C8—C12	120.9 (19)
O1—Pr1—O1^vi^	129.9 (7)	C8—C9—C10	119 (2)
N2—Ag1—N1	174.6 (7)	C8—C9—H9	120.3
N2—Ag1—Ag1^iii^	71.2 (5)	C10—C9—H9	120.3
N1—Ag1—Ag1^iii^	113.5 (5)	C11—C10—C9	119 (3)
C3—C2—C1	118 (2)	C11—C10—H10	120.5
C3—C2—C6	121.2 (19)	C9—C10—H10	120.5
C1—C2—C6	121 (2)	O4—C12—O3	124.9 (19)
O1—C6—O2	124.6 (19)	O4—C12—C8	117.7 (18)
O1—C6—C2	118.3 (19)	O3—C12—C8	117.4 (18)
O2—C6—C2	117.1 (17)	C12—O3—Pr1^i^	150.2 (13)
C4—C3—C2	120 (2)	Pr1—O1W—H1W	100.1
C4—C3—H3	119.9	Pr1—O1W—H2W	126.4
C2—C3—H3	119.9	H1W—O1W—H2W	118.2
N1—C1—C2	123 (2)	Pr1—O2W—H3W	122.7
N1—C1—H1	118.3	Pr1—O2W—H4W	131.7
C2—C1—H1	118.3	H3W—O2W—H4W	105.6
C3—C4—C5	118 (3)		

Symmetry codes: (i) −*x*+1/2, −*y*+3/2, −*z*+1; (ii) *x*, *y*+1/2, −*z*+3/2; (iii) *x*, −*y*+1, −*z*+1; (iv) −*x*, *y*, *z*; (v) *x*, −*y*+3/2, *z*−1/2; (vi) −*x*+1/2, *y*, −*z*+1/2.

### Hydrogen-bond geometry (Å, °)

**Table:**

*D*—H···*A*	*D*—H	H···*A*	*D*···*A*	*D*—H···*A*
O1W—H1W···O2^vi^	0.88	1.79	2.67 (2)	179
O1W—H2W···O4^iii^	0.97	1.68	2.63 (2)	163
O2W—H3W···O2^vii^	1.00	1.77	2.77 (2)	176
O2W—H4W···O2^viii^	0.83	1.95	2.76 (2)	162
O3W—H5W···O1W^ix^	0.82	2.15	2.91 (2)	157

Symmetry codes: (vi) −*x*+1/2, *y*, −*z*+1/2; (iii) *x*, −*y*+1, −*z*+1; (vii) −*x*+1/2, *y*+1/2, *z*; (viii) *x*, −*y*+2, −*z*+1; (ix) *x*, *y*−1/2, −*z*+1/2.
